# A Qualitative Assessment of Community Learning Initiatives for Environmental Awareness and Behaviour Change: Applying UNESCO Education for Sustainable Development (ESD) Framework

**DOI:** 10.3390/ijerph19063528

**Published:** 2022-03-16

**Authors:** Hiroko Oe, Yasuyuki Yamaoka, Hiroko Ochiai

**Affiliations:** 1 The Business School, Bournemouth University, Poole BH12 5BB, UK; 2 Faculty of Society and Industry, The Open University of Japan, Chiba 261-8586, Japan; yamaoka-y@ouj.ac.jp; 3 Department of Plastic and Reconstructive Surgery, Laboratory of Regenerative Medicine, Division of Hearing and Balance Disorder, National Institute of Sensory Organs, National Hospital Organization Tokyo Medical Center, Meguro, Tokyo 152-0021, Japan; ochiroko@gmail.com

**Keywords:** health behaviour, education for sustainable development (ESD), social educational facilities, public living room model, community learning

## Abstract

This study uses qualitative research methods of text mining to elucidate the potential and prospects for community-based learning opportunities for raising environmental awareness and bringing about healthy behaviour change among university students and local residents. In particular, we focus on the importance of community-based learning in raising environmental awareness and inspiring action to support healthy living and harmony with nature. The three groups were triangulated using semi-structured questionnaires to model the ways in which education for sustainable development (ESD) can contribute to the promotion of environmental education in local communities. In order to collect in-depth data, the authors themselves were present at the study sites and collected textual data based on semi-structured questionnaires in a participatory observation framework, where they had a common experience to understand the observations. Analysis was carried out using NVivo12. The two community learning initiatives studied were in Okayama and Tokyo, which are leading ESD policy areas. The two case studies are both university student-led projects that aim to raise environmental awareness in local communities through environmentally conscious behaviour change and the creation of a foundation for healthy living. This study focuses on “youth” and “community” among the five priority areas proposed in the 2015 ESD report and discusses the potential and prospects for community learning initiatives and the triggering of the nudge effect on environmentally conscious behaviour change and health behaviours. The results of the textual analysis with triangulation show that, while policymakers and teachers and leaders driving the initiative acknowledge the importance of ESD in a comprehensive way, their attention is more focused on the design of specific projects and curricula. In contrast, university students engaged in ESD activities rated the social education facilities (local community centres, community learning centres) as “lively” and “motivating”. It was found that there are high expectations for “public living rooms”, which are important as a base for learning to promote healthy and sustainable communities and environmentally conscious behaviour change.

## 1. Introduction

### 1.1. Background of the Study: What to Expect from ESD

ESD consists of learning and activities that aim to create new values and actions by linking social problems with everyday life. In doing so, we are encouraged to think about what we can do to solve them and to take action. Through such experiences, we develop our awareness and ability to act as members of society. ESD is an approach to education that aims not only to provide knowledge but also to help learners reflect on their own values and develop their capacity to participate in the creation of a better society, not just to gain knowledge. It emphasises participatory and experiential learning through problem-solving education and community activities that confront the various challenges to achieving a sustainable society.

ESD is an integrated environmental, social and economic learning approach that includes opportunities to raise environmental awareness and promote behavioural change and well-being for healthy living. While COVID-19 has impacted our lifestyles and mind-set, in anticipation of the post-COVID era, there is a growing interest in the role of ESD as an opportunity to create a more environmentally conscious and symbiotic society.

### 1.2. Research Gap and Aim of the Study

No agreed methodology has yet been established for the educational theories and practices included in ESD. In particular, the contexts on which this study focuses, (1) the potential for ESD to offer suggestions as a representative initiative for community learning and (2) the pathway opportunities for inducing environmentally conscious behaviour and raising awareness of the need for a healthy society, have not been adequately studied.

ESD proponents must continue to seek ways to contribute to the socio-economic problems of their communities and to build sustainable societies, but ESD research has been unable to provide clear guidelines for action. The irony is that ESD research has tended to focus on too many different social issues and that it has become increasingly difficult to share a common understanding and collaborative agenda as a diverse range of participants become involved in contributing to ESD.

However, the lifestyle changes that COVID-19 has brought about and the increased environmental and health awareness that has resulted from being restricted from going outdoors has prompted us to propose a more concrete way of contributing, in the spirit of ESD and in line with the goals of the current UN SDGs.

In particular, the SDGs’ separate Goal 4, ‘Quality of Education’, from Goal 3, ‘Health and Wellbeing’, and it is time to return to the combined perspective of the propositions contained in ESD, namely how community education can contribute to achieving healthy lives and solving a range of other social problems.

Therefore, in order to fill this research gap, this study proposes, through the collection and analysis of qualitative data, a model of the concept of ESD and the way in which ESD activities, as community learning initiatives, should demonstrate the way in which community education can help to build a healthy, environmentally symbiotic and prosperous life.

## 2. Literature Review

### 2.1. Background of ESD and Focus of the Study

To promote ESD, which has recently attracted scholarly attention, it is expected that the mobilising effect of ESD will be experienced in the social educational facilities with which we are familiar. These facilities support the education of people across time and space. The various attempts to make communities more sustainable and economically viable should be seen as the results of periods of reflection regarding the kind of community we originally lived in and the vision that brought us to this point in time. We need to reflect on our learning process [1,2].

ESD is a new approach to environmental education that has been tried and tested. Rather than being an extension of the environmental education that has been implemented so far, it is a type of education that aims to develop the human resources that will be required in a newly formed sustainable society [3]. ESD is based on the values of a sustainable society. Some of its key concepts are connection (including relationships), diversity (including diversity and multi-cultural conviviality), synthesis (including integration and holism) and knowledge (including new knowledge, ethics and public and local knowledge). In other words, ESD is an educational and cultural science that explores the various connections and cycles in the world and emphasises the richness and beauty of the harmony of diversity in the world (which can be seen in the form of sustainability in nature and society). In addition, ESD includes aspects of educational anthropology (philosophy of education), which seeks answers to the question of what it means to be human. From this context, how to change citizens’ behaviour towards a favourable direction could be a target for the ESD agenda.

**Figure 1:**
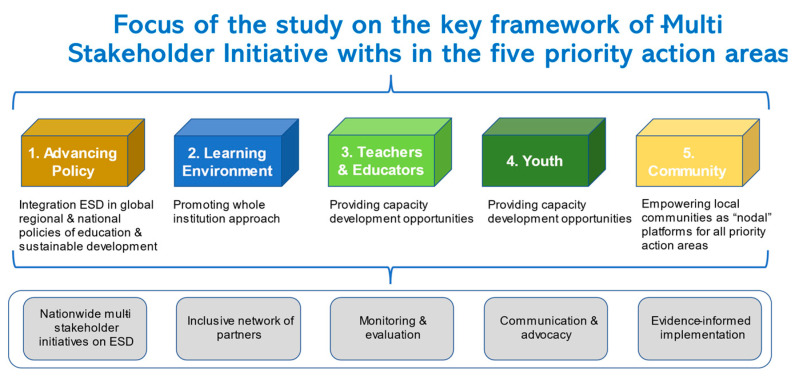
Five priority action areas of ESD (Authors edited and adopted from UNESCO: https://www.oneplanetnetwork.org/sites/default/files/esd_for_2030_one_pager_english.pdf) (accessed on 12 March 2022).

Following the adoption of ESD for 2030, UNESCO published a roadmap of concrete actions to be taken under the framework. The roadmap sets out five priority areas for action: (1) advancing policy, (2) learning environment, (3) teachers and educators, (4) youth, and (5) community (Figure 1). It also identified six priority areas for implementation: (a) implementing ESD for 2030 at the national level (setting up country initiatives), (b) establishing partnerships and collaborations, (c) dissemination to encourage action, (d) tracking emerging issues and trends (evidence-based progress review), (e) leveraging resources and (f) monitoring progress. The main changes from the Global Action Programme (GAP) include the following: an emphasis on the role of education in achieving all 17 SDG goals and a greater focus on major transformations towards sustainable development [4]: In other words, how to learn and share the criticality of increasing environmental awareness and how to increase behavioural change of citizens should be one of the themes for ESD activities.

### 2.2. Role of ESD in Enhancing Environmental Awareness and Behavioural Change

The practical aspects of ESD recognise the importance of building collaborative networks that lead to the creation of a sustainable society, and the characteristics of this approach (the practical theory of ESD) can be seen in its use of a community of practice approach to the search for knowledge. It emphasises a holistic search comprising the environment, economy, society and culture.

ESD is, therefore, an approach that integrates scientific and social scientific knowledge with local and practical knowledge based on network and co-value creation. It should be noted that ESD scholars are trying to construct a theory of human development as required by the new society based on social network interventions [5]. With its characteristics, ESD can be applied not only to the study of the natural environment but also to the study of social development in connection with the exploration of various social issues and challenges, such as combating environmental issues, enhancing mutual respect for diversified cultures, and poverty eradication. Through this ESD lense, it can be expected that we can develop actionable suggestions for communities on how to increase public health using the learning process of improving our lifestyles with respect for environmental values [6].

Schools function as places of public education, but learning is not limited to the interaction of people involved in the community and the motivation for spontaneous and interactive learning that is fostered by this interaction. From the standpoint of seeking ways to cope with the various social problems experienced in everyday life, attention has been drawn to education in the broadest sense, which is produced by a variety of situations, aspects and people [7]. The theme of teaching and learning to promote public health, which is the focus of this study, has the potential to make a significant contribution to the eternal imperative of maintaining and improving people’s health in building sustainable communities.

### 2.3. ESD and SDGs: How to Fuse Goal 3 & 4

As a result of the community-wide ESD project, the number of participating organisations has increased, including schools, community centres, civic groups, universities, businesses and government agencies. It is timely to consider how social educational institutions, such as libraries and community centres, have contributed to ESD, tackling public health issues and others in communities [8]. ESD has been identified not only as one of the targets but also as a contributor to the realisation of all 17 of the SDGs, as confirmed by the UN General Assembly at its 74th session. ESD, which prepares people for a sustainable society, contributes to the realisation of quality education. This is essential for meeting the SDGs, which is made clear in the new international framework for ESD—Education for Sustainable Development: Towards the Realisation of the SDGs: ESD for 2020–2030—adopted at the 40th UNESCO General Conference in 2019 [4]. 

### 2.4. The Role of Social Educational Facilities in Communities

#### 2.4.1. Community-Led Consortia to Realise Sustainable Society

Long before the UN explicitly proposed ESD policies, social educational facilities—the origin of such policies—were common and accessible to local residents and visitors, and many have visited these facilities at least once to learn about their culture and history and determine a future path for sustainable development. Therefore, it is expected that an ESD strategy with community centres and other social educational facilities should be easy to understand and feasible for policymakers, users and others [1,9].

According to the resolution of the 57th session of the UN General Assembly in December 2004, the decade beginning on 1 January 2005 was to be known as the UN Decade of Education for Sustainable Development. UNESCO, which was designated as the international driving force during this decade, presented the International Decade of ESD. In 2004, at the 59th session of the UN General Assembly, UNESCO published a draft titled ‘International Decade of ESD Implementation Plan’ [10]. The plan states that the objectives of the International Decade of ESD are to promote a broad understanding of the role of education and learning in humanities, including citizens’ health and well-being, and to promote networking and exchange wisdom and experiences to pursue the targeted aim [11].

#### 2.4.2. Societal Actors Collaboration to Contribute to Communities

ESD can be implemented in a variety of ways in accordance with the circumstances of each community and individual. As the UN resolution declares, Japan—under the leadership of the central government, local governments, public institutions, NPOs, residents’ groups and businesses—is promoting sustainable development based on socio-economic issues on the agenda.

Several regions in Japan have strategically promoted ESD activities using their own individual plans. Among them, Okayama is one of the most active areas as a model for Japanese ESD. The Okayama ESD Promotion Council was set up with several universities. Together with local governments and boards of education, they seek to implement practical ESD initiatives in promoting public health with collaborative activities involving schools, higher education institutions, and citizen groups [12]. Since then, following the SDGs’ slogan ‘leave no one behind’, various efforts have been made locally, nationally and globally to realise the 17 goals and 169 targets for 2030. The Okayama Project has been moving into a concrete phase, during which it is necessary to define the ideal state of Okayama with policy aims and objectives to enhance community health with healthy behaviour among citizens [13].

Ref. [14] discussed the impact of ESD in enhancing collaborative approach to achieve sustainable community, whereas [15] examined the outcome of ESD implementation from various countries’ case studies [16], in developing education for sustainable development (ESD), based on the fact that universities have the function of learning and teaching themselves, participating in the development of teaching materials from other disciplines, involvement in the development of learning methods, and responsibility for sustainable development science, as well as the function of promoting the process of ESD This is actually a very important point. This is, in fact, an argument that encompasses a very important point. Learners and teachers who have a systematic understanding of social and natural processes, as well as expertise in local cultures and traditions, are the best people to drive ESD when it comes to the themes of inducing environmentally conscious behaviour and promoting community health. As the next generation of ESD leaders, university students are an important source of capital, as well as a hub for engaging university faculty and a diverse range of stakeholders (community agencies, R&D, consultants, businesses, etc.). The patterns of curriculum change processes that embed the university are also important [17].

Due consideration of the complexity and diversity of the challenges associated with the implementation of ESD also implies the importance of the strategic distribution of the resources of universities and other community stakeholders to ensure that the contribution of academics is optimised in the promotion of the ESD process. In order to promote environmental citizenship education, it will also be important to strengthen the links between ESD and science education and to create and operationalise initiatives that embody the spirit of ESD [18].

### 2.5. Social Capital as a Basis for Sustainable Communities

The three key areas of sustainable development are society, the environment and the economy, with culture as a fundamental element. The concept emphasised in this paper can be interpreted as placing culture with its diversity as the basic underlying concept and then emphasising the need to work together in the three aforementioned areas.

However, regarding the various socioeconomic problems that sustainable development policies aim to address by offering resources for each actor in the social network and through a collaborative system, it is important for the key actors to function as the coordinators and leaders of the sectors. In relation to this, ref. [1] considered the possibility of mobilising social capital and focused on the existence of actors who can play a catalytic function in strengthening cooperative behaviour, for instance, realising healthy communities requires interdisciplinary approach, not only from the medial sciences, but also from social sciences, community development, community design, social marketing, and so on. It is critical to integrate the humanities and social sciences to co-create community value such as healthy behaviour. Ref. [19] suggested that perspectives of social capital in supporting resilient community with healthy members could be a useful guidepost for further critical discussions.

Moreover, in light of the environmental education framework, the importance of the role that ESD has to play today is highlighted. For example, ref. [20] focuses on the effect of connectedness in environmental learning and discusses the direction of education and the functioning of teachers in promoting the importance of environmental behaviour, while [21] suggests the use of interactive games in environmental education. Ref. [22] also emphasises that learners’ opinions and evaluations should be closely captured to ensure a loop of reflection and improvement in the curriculum of environmental education.

### 2.6. Main Research Questions of This Study

Confronting the social challenge of social network interactions to raise environmental awareness and support healthy behaviours, the research scope is firstly organised to provide clear proposals and signposts to this end.

1. It is a serious policy challenge for today’s global citizens to improve public health and promote behavioural change among citizens through comunity learning, with the guiding principle of achieving the SDGs. It is timely to consider how this research can contribute to this challenge through the lens of ESD.
2. Education should contribute to the creation of a symbiotic society and the health of the planet. In considering the health of the local and global communities in which we live, rather than introducing multiple structural strategies such as regulatory policies and sanctions, it is important to appeal to people’s psychology, encourage cooperative behaviour, avoid social dilemmas, and solve problems from a medium- to long-term perspective, which is also an effective perspective from the perspective of the sustainability of problem solving [23].
3. Education should contribute to the maintenance of cultural diversity, which should be the basis of the three elements of society, economy and environment and lead to the creation of communities with stable and flexible cultures. As [24] argued, it is the interrelationships that enable people to unite in community, committed to each other, and that are the main force for weaving the social fabric. It is important to promote collaboration among community actors to increase co-existing values, public health, and well-being in communities. From this perspective, social and educational institutions may function as hubs for creating such commitment, exchanging information and promoting behavioural change.

Therefore, through the analysis of the qualitative data collected by qualitative methods, the research questions that this study aims to identify are defined as follows

RQ1: How do community-based learning initiatives in the spirit of ESD contribute to environmentally conscious behaviour change and healthy lifestyles and communities, and how do participants in ESD activities value this?
RQ2: What are the key elements in promoting collaborative and participatory learning initiatives involving a diverse range of citizens to raise environmental awareness and promote behaviour change?
RQ3: Can social education facilities, as places that foster ESD, act as catalysts for enhanced learning outcomes for participants [25] and serve as the basis for environmentally conscious behaviour change and healthy citizenship?

## 3. Methodology

### 3.1. Outline of the Research Method

Using an ethnographic approach, this study examines how community learning ESD initiatives influence people’s environmentally conscious attitudes and behaviours and how they contribute to healthier and happier lives and societies. As stated in RQ3, the study will examine the impact of the initiatives on university students, teachers and leaders who participate in ESD activities through social education facilities and on ESD-related policymakers and local authorities.

The research focuses on initiatives in Okayama Prefecture and Tokyo, two of the most advanced ESD regions in Japan, and uses an observational approach: the actions and behaviours of university students and teachers were observed and recorded, and semi-structured questionnaire interviews were conducted. In addition, to triangulate the outputs from the two categories, a third category of policymakers was invited for interviews, in both regions, to collect primary data from each of the three groups. The core research participants were university students who will become future ESD leaders, and this study explores the possibilities and challenges for their development and learning as ESD leaders by examining their interrelationships and interactions [26], as well. In addition, we triangulated qualitative data from three groups of participants, including ESD leaders and policymakers, in order to provide valid and unbiased suggestions [27].

The participant-observation method used in this study is a special form of the nature-observation method, which ensures a high degree of ecological validity because the researcher places himself in the field under study, observes it from within its context, and has a common experience to understand the observations. In cultural anthropology, and especially in sociology, this method aims to deepen the understanding of the whole through the researcher’s senses by having the researcher present in the field of observation, establishing a trusting relationship with the target group and acting as a member of the group [28]. In concluding the process of participant observation by the researchers, the primary data for the actual textual analysis was collected meticulously from the respondents based on a semi-structured questionnaire, recorded, and transcribed, and the data were coded using NVivo12 for analysis.

Prior to the implementation of this study, a preliminary survey was conducted twice a month for one year in order to identify research cases and develop a research plan. In addition, we actively participated in ESD activities. The observation records were carried out with the aim of reproducing the atmosphere of the field as much as possible and with a constant awareness of rapport with the observed subjects, including during self-analysis, and, at the end of the process, interviews with semi-structured questionnaires were carried out. Textual data were collected from (1) 15 students, (2) 3 teachers/leaders, and (3) 5 policymakers (46 participants in total from the two regions) for each participant at both sites for each initiative, and all data were anonymised using ID codes.

### 3.2. Research Targets

In this paper, the phase of consciousness of the above three groups of subjects towards ESD activities held in (a) a community center in Okayama and (b) a youth club in Tokyo is captured and analyzed. In order to make the modelling of community learning initiatives [29] accessible and feasible to initiative designers, project conveners, participants, and policymakers and other stakeholders for future ESD development, both (a) and (b) were social education facility-based activities that extended into the local community sites, and each had the following ESD themes.

**Figure 2:**
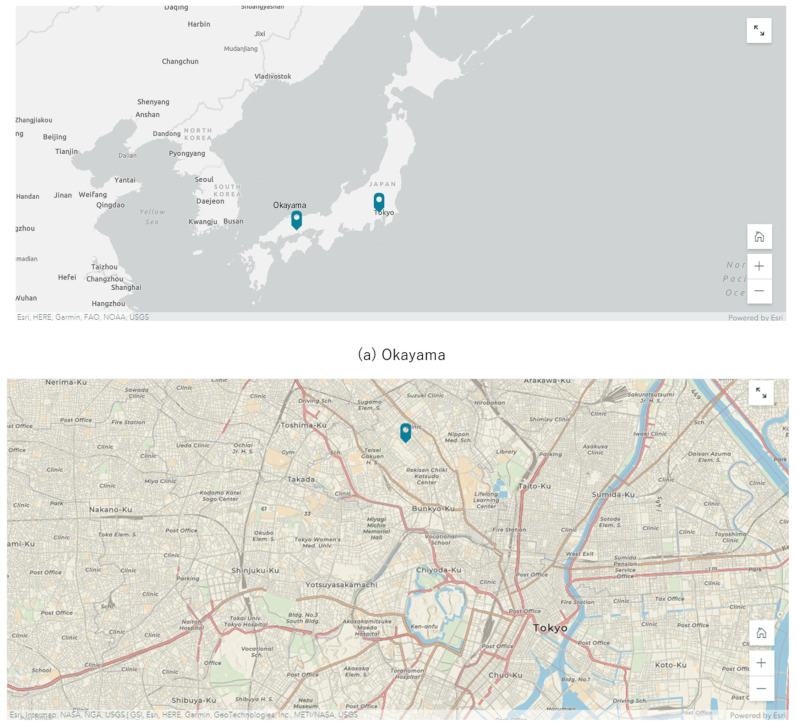

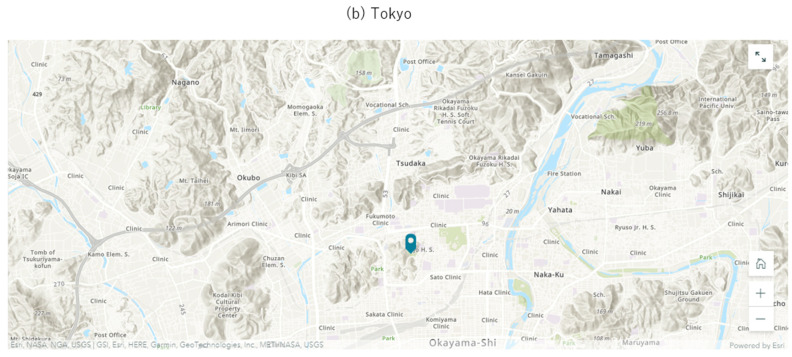
Outline of two ESD activities: Okayama (**a**) and Tokyo (**b**). (**a**) Kyoyama, Okayama, ESD initiative ‘Understanding the landscape and geography of the mountains and rivers of Okayama and creating a society in harmony with the environment. (**b**) Hakusan, Tokyo, ESD initiative ‘Learning opportunities by enhancing Tokyo 2020 legacy in metropolitan area’.

As Figure 2 shows, the two ESD activities in the two regions used different approaches to learning about environmentally conscious behaviour change and healthy communities, based on different community contexts and conditions. Project (a) was based at a social education facility in the northern part of Okayama City, close to the mountainous area, and involved learning from each other about the importance of coexistence between nature and human beings and creating action guidelines to pass on to future generations. On the other hand, (b) was a project led by university students, based in the heart of the capital city of Tokyo, to implement an urban environmental education programme based on the legacy of the Tokyo Olympics and Paralympics, which were postponed until 2021.

### 3.3. Analytical Scnenario—Case Studies

In 2011, citizens and stakeholders working on ESD in Okayama Prefecture developed the “Okayama Model” in order to promote ESD through a consortium approach involving local stakeholders in addressing community issues. The framework includes the following five points or “Okayama Indicators”.

(1) There should be a place for dialogue among various people and organizations.
(2) The city serves as the secretariat and provides stable management involving all relevant parties.
(3) The presence of a full-time ESD coordinator
(4) The community centre becomes the hub for ESD promotion in the community
(5) Universities taking on a leading role in ESD in the region.

In this study, we interviewed university students, who are the leaders of ESD, about how they understand and appreciate their functions as executors and practitioners of environmental education. Based on Okayama Indicators 1–5, the interviews were conducted in a friendly atmosphere, including icebreaking, to discuss the importance of raising environmental awareness, spreading awareness, and changing behaviours among the younger generation. The duration of the interviews was about 30–45 min per interviewee.

### 3.4. Findings and Analysis

#### 3.4.1. Okayama Initiative

Taking as a case study the ESD activities conducted at K Community Center, one of the leading ESD activity centers in Okayama Prefecture, we examined the possibilities and pathways for local university students to lead local ESD activities and contribute to community building through modules on environmental awareness and behavior change, health care, and the importance of living in harmony with nature. It is clear from the above five indicators that the involvement of groups and organisations with a wide range of backgrounds and areas of expertise is of central value in the development of human resources capable of addressing local issues from an ESD perspective.

**Figure 3:**
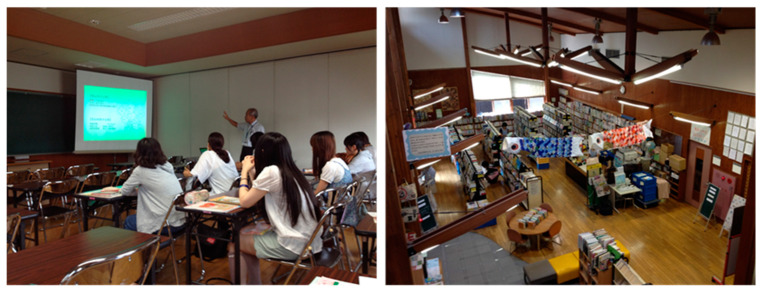
Photos of ESD activity scenes (Okayama case).

Another distinctive feature of the Okayama Prefecture initiative is the explicit positioning of community halls as centres for ESD promotion, with the expectation that they will function as public living rooms for the community and as places for interaction with a wide range of people and organisations. Universities are strongly advocated for as supporters of local problem-solving. It is strongly advocated that, in the communities covered by this study, young leaders are leading intergenerational exchanges on the development of environmental education programmes based on the characteristics of their hometowns, assuming geographical environmental assets such as mountains and rivers (Figure 3). 

A case study from Okayama Prefecture points to the fact that ESD as an extracurricular activity for university students provides an important opportunity to improve people’s awareness of environmental issues in the region and contributes to the development of teamwork skills, especially among university students who are expected to perform driving functions there [30]. Such ESD activities can also contribute to the acquisition of leadership skills among university students, which can be expected to have a positive impact on the future employability of participating university students.

#### 3.4.2. Youth Club Activities in Tokyo

From the observation of the youth club in Tokyo, it has been found that ESD-related activities conducted at a community centre can be a trigger for nurturing university students’ capability and leadership through team-learning in the context of ESD. This outcome is supported by discussions presented by [31], who suggested that it has been critical to focus higher education on enhanced students’ critical thinking and ideas in the context of contributing to communities. Educational institutions such as universities should be encouraged to develop partnerships with multi-sectoral stakeholders in the context of ESD. A platform for collating transdisciplinary partnerships is needed in the community.

The theme of the ESD project in Tokyo was to propose ways to contribute to the creation of a sustainable regional, national, and global community based on the intangible spirit and tangible legacy of the Olympic Games. It was an initiative to propose ways of contributing to the development of a community that is healthier, more harmonious with nature, and free from environmental challenges and poverty, in a metropolitan area with an influx of diverse immigrants and a pluralistic society.

## 4. Findings and Analysis

### 4.1. Text Mining of Students’ Views

**Figure 4:**
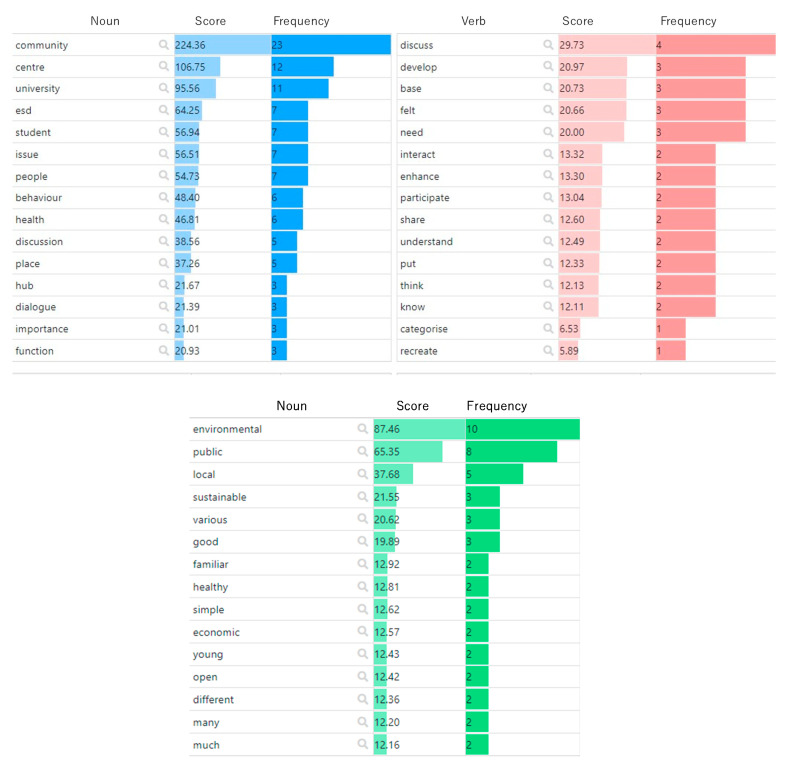
Frequent words by part of speech.

The results of mining frequent words by part of speech from the students’ utterance data are shown in Figure 4. Some words with a high frequency of occurrence, but with a diluted meaning and little importance, are mixed in the transcribed data set. In other words, in text mining, it is not possible to grasp the characteristics of a text by simply ranking the number of times it occurs. At the same time, the TF-IDF method was applied as the logic for the extraction of feature words that fit the purpose of the study [32].

**Figure 5:**
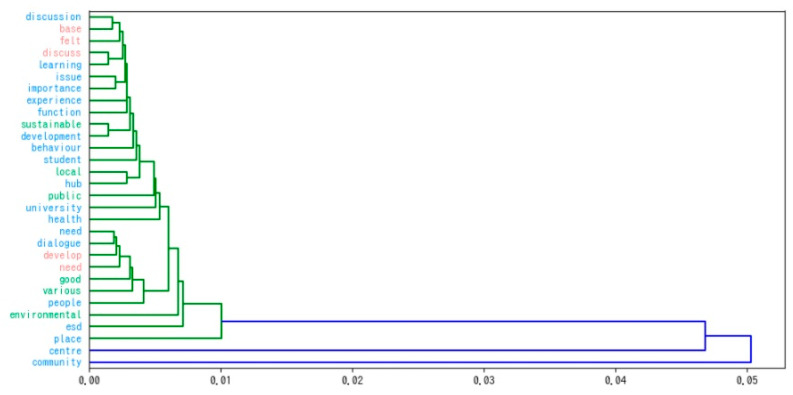
Hierarchical clustering.

Hierarchical clustering illustrates the process of grouping words with a similar tendency to appear in clusters (Figure 5), in order of similarity. Similar to the tree diagram used in biological evolution, similar words branch closer together (on the left), and dissimilar words branch further apart (on the right), giving a hierarchical view of the grouping of words with similar tendencies.

The proximity (similarity) between words/clusters when grouping clusters is determined by the position of the vertical lines grouping the clusters: the further to the left, the closer; the further to the right, the further away. This figure shows that the clusters are concentrated on the left-hand side of the graph; the trends are relatively similar, and the students interviewed are in general agreement that they perceive ESD in a similar way and that they evaluate ESD and social education facilities as activity sites according to generally agreed values. The colours of the hierarchical clustering tree diagram provide an intuitive picture of students’ attitudes, as similar words are grouped together in the same colour.

From the database, a co-occurrence network map was also developed to demonstrate word-to-word relationships. We used KH Coder, a text mining software, for this task to conduct the morphological analysis; word extraction, co-occurrence mapping, and hierarchical clusters [33,34]. For a co-occurrence map, words closely associated with each other are connected with lines, especially bold lines, which imply which are more closely associated with each other as subgraphs through colour coding. A co-occurrence map was developed based on the Jaccard coefficient indices that demonstrate close relationships among words [35]. Following co-occurrence mapping, Ward’s hierarchical clustering method with Jaccard coefficients was applied to the dataset to determine the degree of word-to-word co-occurrence and create clusters [34].

**Figure 6:**
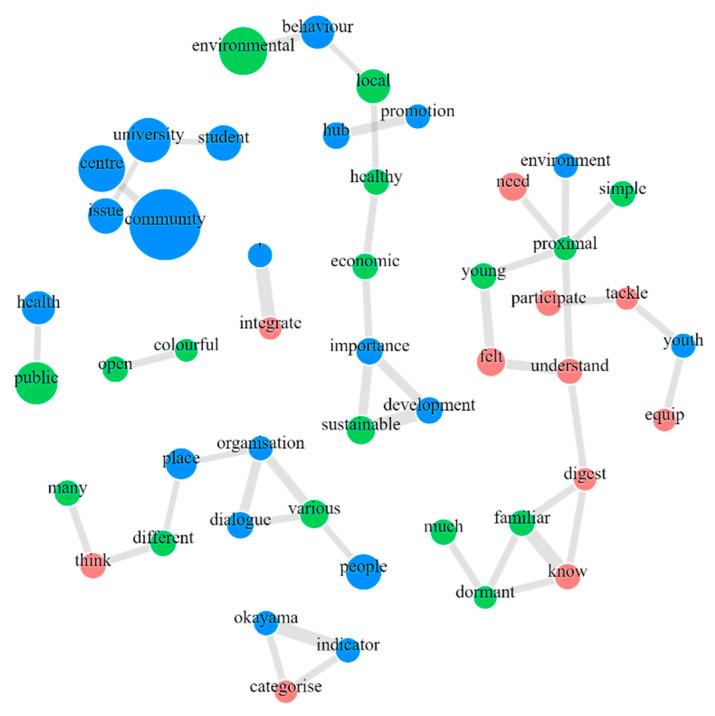
Co-occurrence map.

Figure 6 shows the co-occurrence map. It can be seen that the students recognise the importance of the younger generation in driving the activities to contribute to environmental issues and to have a deeper understanding of where problems lie. At the same time, they also acknowledge the criticality of economic growth for the sustainable development of society.

**Figure 7:**
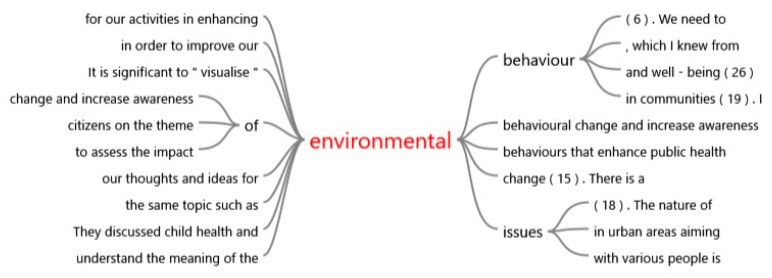

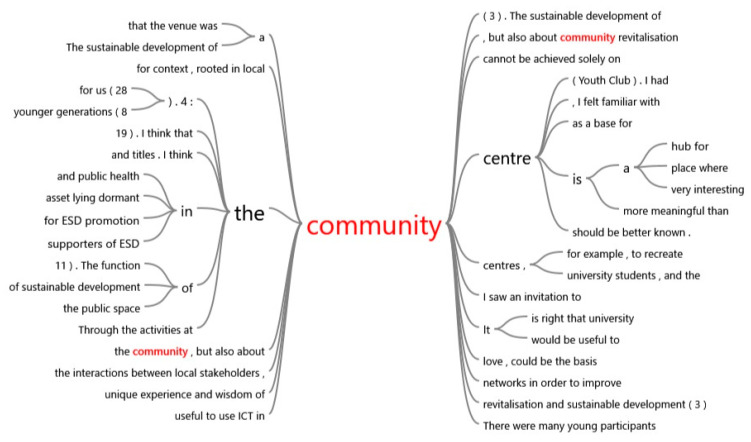
Word tree for ’environment’ and ‘community’.

An exploratory review of the textual data from the interviews with a total of 30 participants in the ESD project provides an overview of how the words ‘environment’ and ‘community’, the core themes of the study, are perceived and evaluated by the students (Figure 7).

Exchanges at the social education facilities have led to an awareness of socio-environmental issues and challenges, which the students themselves enjoy. By learning from each other, they have also become more aware of their role as promoters of ESD and have shown a willingness to participate actively.

### 4.2. Text Mining of Teachers/Leaders and Ppolicy Makers

**Figure 8:**
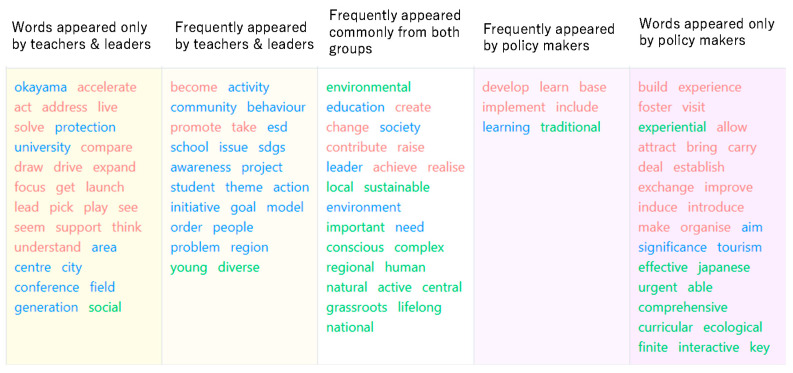
Comparative overview of frequent words of teachers, leaders, and policymakers.

Figure 8 shows the results of text-mining a summary of the comments of the lead teachers and local government ESD policymakers. Both teachers and policymakers are responsible for guiding and mentoring students, who are expected to be substantial contributors and drivers of ESD, as well as developing guidelines for ESD practice at a higher level. Therefore, the word outline in Figure 8 can be expected to encompass the future vision of ESD, its challenges, and to suggest ways in which ESD can contribute to inspiring healthy living and environmentally conscious behaviour, which is the subject of this study.

Although both teachers and ESD policy-makers have the responsibility of guiding students in ESD practice, there are interesting differences in what they say, as shown in Figure 8. In other words, the teachers, who are closer to the students, are more focused on helping them understand the environmental problems of their communities and the importance of environmentally conscious behaviour in the context of closer case studies, whereas the policymakers are more concerned with how to translate higher-level concepts, such as the importance of circular economy and ecological values, into ESD initiatives on the ground.

**Figure 9:**
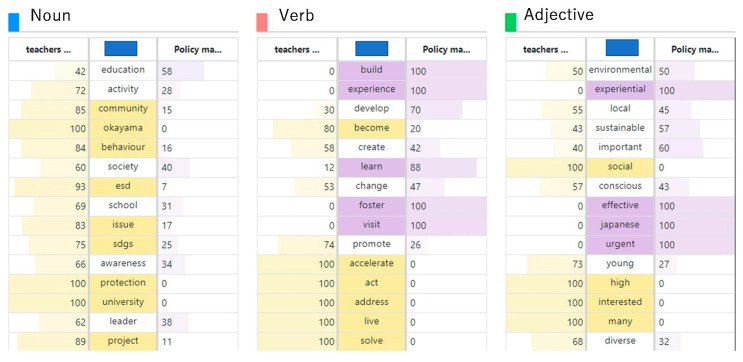
Comparative word analysis for two groups.

**Figure 10:**
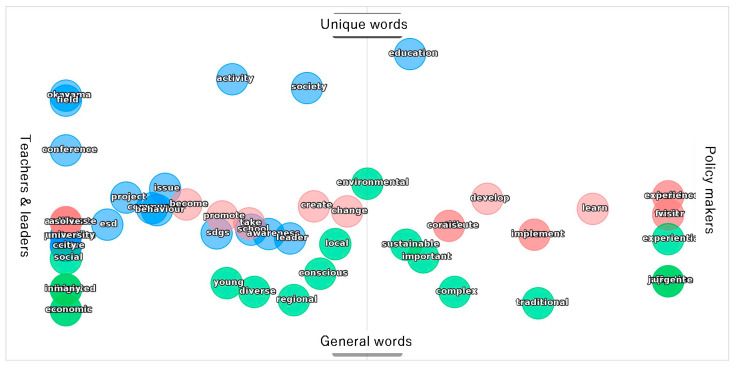
Feature word map.

Figure 9 shows a comparison of the frequency of the utterance words in both sides. From Figure 10, the different interests and behavioural principles of the two groups can be clearly seen. In particular, the adjectives that appear frequently in the statements of the two groups show that policy-makers are more interested in how to embed ESD in environmental issues, which are urgent issues for Japan as a whole, from a higher perspective, and how to link them to concrete implementation plans.

To review more in details of the two groups’ perceptions, Figure 10 was developed. This figure shows a two-dimensional mapping of which document each word occurs in more often and how characteristic the word is of the document. The uppermost word indicates that it is a characteristic word of this document, while the lowermost word indicates that it is a general word that can appear in any document.

The feature word map shows that both teachers and policymakers are speaking more about ESD from a social problem-solving perspective, but that policymakers are particularly concerned, from an educational perspective, with mechanisms and interventions to promote healthy living and environmentally conscious behaviour in communities and citizens, and that teachers are more concerned with planning and implementing specific projects to support student learning.

### 4.3. Discussion

#### 4.3.1. Differences between the Three Perspectives for ESD Implementation

Whereas teachers and policymakers tend to take a bird’s-eye view of ESD and focus on formulating basic guidelines and frameworks for programmes, the younger generation who actually participate in ESD activities tend to enjoy the learning process and are more motivated by spontaneous learning and action. In particular, the process of experiencing individual environmental and local contextual issues, deepening awareness and sharpening consciousness through interaction with others, and returning to their home base of social education facilities to further verbalise and share experiences and consider next actions is seen as one of the most important aspects of ESD activities.

Community-based ESD initiatives are likely to function effectively through a three-ring system that supports the aspirations of the young generation of front-line practitioners and implementers by creating the environment, providing the tools and space for practice, and setting an agenda that the participants will review, assess, and feedback for the next step of activities.

#### 4.3.2. Community Centres as a Place for Loose Links and Information Sharing

**Figure 11:**
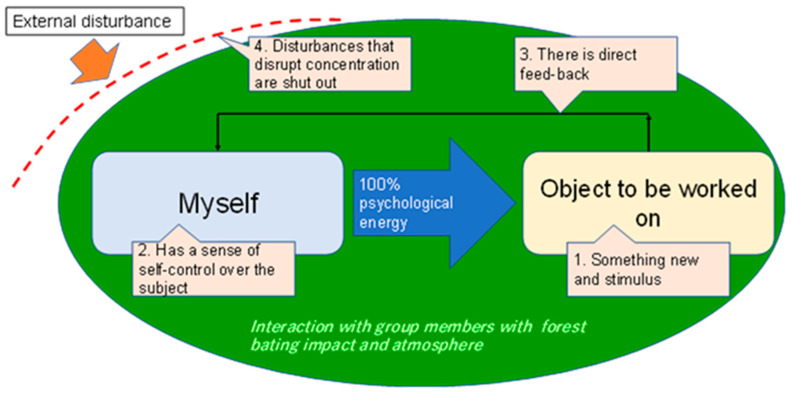
‘Flow’, with an impact on learners’ concentration.

One guidepost for the effects of concentration and arousal in learning is the concept of flow, which has been proposed in the field of social psychology and has been developed and applied in various fields. Flow is one of the factors that contribute to the success of learning situations, and it may be applied to the framework on the induction of environment-oriented behaviors, which is the subject of this study. Originally, flow is described as a state of being ‘in the zone’, which is achieved when the learner is cut off from the external world and concentrates on the task at hand [36]. In other words, flow theory is argued to be a state produced by being cut off from the outside world, eliminating noise, and concentrating on a single point (Figure 11).

However, according to the results of the text mining, in the process of learning about the importance of environmentally conscious behaviour and the promotion of health through collaborative ESD, it was often stated that loose connections, lively discussions, and spaces for such discussions are more effective for the implementation of ESD projects. Many of the participants claimed that the community centres were important ESD hubs and that they were grateful for the provision of such spaces. They drew on their own experiences of learning about ESD, put it into their own contexts, and tried to weave solutions through inner reflection. In doing so, they expressed that the community centre is an effective place for learning and interaction with other participants and for spreading awareness.

While some participants did indeed express a positive view of the concentration suggested by flow theory, it is interesting to note that they were more positive about open and flexible information dissemination and the inflow of information from outside. They valued the inclusion of unanticipated information and the development of unplanned discussions. In this sense, the community centres in ESD projects seem to value more the gradual influx of information and consequent discussion through external connections. Rather than being a place of concentration, the community centres seemed to function more as a catalyst for mutual learning, in line with the ESD theme of environmentally conscious transformation and community health.

In other words, participants recognised that the ‘public living room’ of the community centre effectively brought people together and facilitated interaction through a loose connection with the outside world and the associated freedom for people to come and go [1]. In particular, the university students who participated in this study seemed to accept the input of diverse stakeholders as an ESD-type learning infrastructure that contributes to environmental conservation and environmentally conscious behaviour and to be motivated and responsible to pass on these narratives to others through community-based discussions. A sense of responsibility also seems to have emerged [37]. At the same time, it is noteworthy that the trust between stakeholders in community learning is based on continuous learning, such as in ESD activities [38].

### 4.4. Proposal of a ‘Public Living Room Model’ in Enhancing Environemntal Behaviour and Contributing to Communities

**Figure 12:**
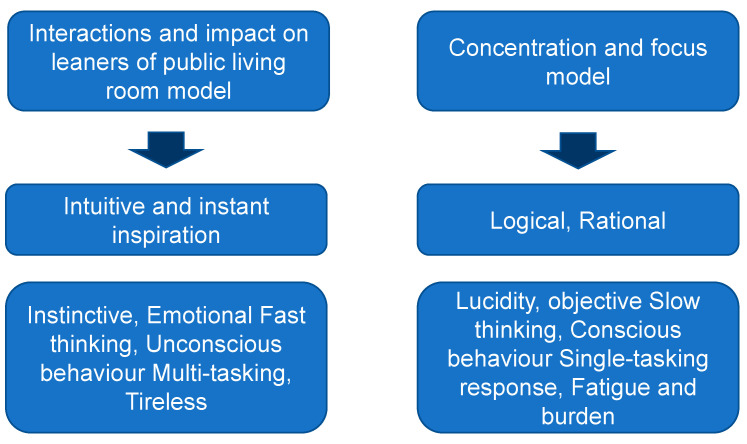
A comparative image between the ‘public living room’ model and ‘focus model’.

In this section, based on the data obtained and the results of the analysis, we propose a ‘public living room model’ for community learning enhancing people’s environmental awareness and leading to behavioural change. The public living room is important as a place where problems are identified, information is shared, and people decide to change their behaviour. The buzz of the surroundings, the occasional informal chat with the neighbouring teams, and the possibility of new discoveries excited the participants. It is not a silent, laboratory place of learning but rather a place where moderate interaction occurs, and people can work independently and spontaneously on their own projects (Figure 12). The outline of the comparative view indicates that student-led ownership of discussion and activities contribute to mutual learning in enhancing environmental awareness and behaviour change.

The implications of this study are novel and unique. There has been a certain amount of research on environmental education and ESD, as already discussed. In the past, when considering the functioning of ESD, there has been integration and collaboration with school curricula [13,39], as well as different attempts to redefine environmental education in the context of ESD [40], and the guidelines and suggestions of studies, such as on the effectiveness and significance of learning together in communities [41]. Building on these insights, the present study showcases the application of flow theory, which has attracted much attention in learning, and its counterfactual implications, and explicitly highlights the significance of learners’ interactions in the public living room model. The models and concepts proposed here are arguments that are distinct from the claim to confront learning themes in a concentrated way.

Ref. [42] discussed that, in an interdisciplinary course on ESD, empirical research shows that learners suggest that in-class active learning exercises make the lessons more engaging and that they appreciate the flexibility of learning together through reflection practices and in-class mini-lectures. They also emphasised that flipped classroom teaching stimulates active and reflective learning motivation for ESD. In line with their discussion, [43] argued that anthropogenic environmental changes, in particular the sustained loss of biodiversity and the decline of the world’s forest resources, require comprehensive social change towards sustainable behaviour and that ESD will increasingly be responsible for sustainable decision-making, cooperative participation, high levels of commitment, and motivation to protect the environment.

A comprehensive approach to education for sustainable development is the subject of this study, but it must be said that, without a combination of knowledge acquisition and emotional drive, it is difficult to foster environmentally friendly behaviour.

In order to organically mix this secure and accurate knowledge, the interdisciplinary wisdom and willingness to learn fostered through interaction with diverse stakeholders and the sensitivity that serves as an incentive to act in an environmentally conscious manner is essential to having a loose connection and an open mindset of mutual respect and listening, as suggested by the public living room model, in order to use them as a major force for change.

Classroom discourse is an integral part of the teaching and learning process in all disciplines, including science, and the public living room model, as suggested by the text mining results, appears to have the potential to further motivate and enhance participants’ learning through loose connections and flexible information exchange in the ESD learning process. As [44] argued, the richness of discourse analysis methods developed by science education researchers can provide useful tools for studying, teaching, and learning processes in science classrooms, including ESD education, and in learning spaces in the broadest sense, including ’public living rooms’.

Ref. [45] also argued that human capital is the total accumulation of an individual’s social, academic, psychological, market and internal value capital. As discussed, university students, under the guidance of teachers and leaders, voluntarily confront ESD issues in the space of mutual learning in the ‘public living room’ and participate in the process of proposing guidelines for action and solving problems. Lifelong learning through ESD activities that raise environmental awareness and inspire environmentally conscious behavioural change will have the effect of amplifying lifelong learning opportunities for different generations in the community by providing loose connections and learning opportunities in civic life and enhancing the interaction of the complex human capital inherent in each individual.

Let us now conclude our discussion of the public living room model illustrated here and its implications for ESD, in the light of the three RQs that were initially developed.

RQ1: All three groups, university students, ESD leaders, and community stakeholders focused on in this study recognise and are proud to be part of community-based learning initiatives in the spirit of ESD that contribute to environmentally friendly behaviour change, healthy living, and community development.
RQ2: In promoting collaborative and participatory learning activities involving diverse citizens to raise environmental awareness and promote behavioural change, the gradual linkages and flexible exchange of information and learning among people brought about by social education facilities rooted in local communities were found to increase participants’ motivation to learn and boost ESD impact on local communities and citizens.
RQ3: Together with the responses to RQ1 and 2, it was confirmed that social education facilities, as places that foster ESD, can be a catalyst for participants’ learning outcomes and a foundation for environmentally friendly behaviour change and healthy citizenship. In particular, as the public living room model suggests, the potential for the further utilisation of social education facilities, which, unlike school facilities as places for concentrated learning, are loose networks of connections, should be further promoted and positioned as local assets to be utilised.

## 5. Conclusions

### 5.1. Contribution

Using qualitative methods, this study critically assessed the possibilities and prospects of the UNESCO-proposed ESD framework for raising environmental awareness and changing the environmentally conscious behaviour of citizens in local communities, thus creating a healthy society. Triangulation was used to identify university students who are expected to function as the next generation of ESD leaders, teachers who are in the position of supervisors of ESD activities to mobilise them, and policymakers who are implementing relevant policies and schemes in the communities.

We compared and analysed textual data collected from three groups. We modeled and proposed avenues for community learning initiatives for environmental awareness and behaviour change. From the research, a public living room model was proposed as the original outcome of the study, suggesting the possibility of stimulating environmental behaviour through loose cooperation, information sharing, and the propagation of awareness. While the importance of immersion in intensive learning is emphasised in flow theory, this study revealed that the buzz of laughter, a more relaxed environment, and sometimes noisy interferences had a catalytic function and were perceived by the participating students as a major motivating factor in “promoting gradual learning cooperation” in order to recognise the location of environmental problems in the community, share awareness of the problems, and drive action to solve them.

### 5.2. Limitations and Further Research Opportunity

In this study, observations were made in two ESD activity cases led by university students using participatory methods and text data collected from 46 participants based on a semi-structured questionnaire were analysed by text mining. The results of the data analysis suggested the significance of the functions of local social education facilities in ESD, especially the potential for health-oriented behaviour change among university students, and the pathway to increased awareness of public health.

However, it was recognised that further research is needed to further elaborate and generalise the conclusions and models obtained in this study. Two opportunities for future research are envisaged. First, more qualitative data from multiple ESD action cases should be collected to validate the findings of this study. Secondly, to make more concrete recommendations, a survey should be conducted, quantitative data collected from stakeholders, and a conceptual model and scale proposed and validated to contribute to further research and discussion.

## Data Availability

The data are not publicly available due to this data was obtained under conditions that are not intended to be published.
